# The Cytotoxic Role of Intermittent High Glucose on Apoptosis and Cell Viability in Pancreatic Beta Cells

**DOI:** 10.1155/2014/712781

**Published:** 2014-03-17

**Authors:** Zhen Zhang, Jing Li, Lei Yang, Rongping Chen, Rui Yang, Hua Zhang, Dehong Cai, Hong Chen

**Affiliations:** ^1^Department of Endocrinology, Zhujiang Hospital, Southern Medical University, Guangzhou 510282, China; ^2^Department of Endocrinology, Nanshan Affiliated Hospital of Guangdong Medical College, Shenzhen 518052, China; ^3^Department of Nephrology, Zhujiang Hospital of Southern Medical University, Guangzhou 510282, China

## Abstract

*Objectives*. Glucose fluctuations are both strong predictor of diabetic complications and crucial factor for beta cell damages. Here we investigated the effect of intermittent high glucose (IHG) on both cell apoptosis and proliferation activity in INS-1 cells and the potential mechanisms. *Methods*. Cells were treated with normal glucose (5.5 mmol/L), constant high glucose (CHG) (25 mmol/L), and IHG (rotation per 24 h in 11.1 or 25 mmol/L) for 7 days. Reactive oxygen species (ROS), xanthine oxidase (XOD) level, apoptosis, cell viability, cell cycle, and expression of cyclinD1, p21, p27, and Skp2 were determined. *Results*. We found that IHG induced more significant apoptosis than CHG and normal glucose; intracellular ROS and XOD levels were more markedly increased in cells exposed to IHG. Cells treated with IHG showed significant decreased cell viability and increased cell proportion in G0/G1 phase. Cell cycle related proteins such as cyclinD1 and Skp2 were decreased significantly, but expressions of p27 and p21 were increased markedly. *Conclusions*. This study suggested that IHG plays a more toxic effect including both apoptosis-inducing and antiproliferative effects on INS-1 cells. Excessive activation of cellular stress and regulation of cyclins might be potential mechanism of impairment in INS-1 cells induced by IHG.

## 1. Introduction 

Diabetes is becoming a serious worldwide threat to human health and rises rapidly in China recently. Pancreatic *β*-cell mass should be dynamic and can respond to physiological and pathological variations in metabolic demand on insulin production. But this ability of the endocrine pancreas seems to be attenuated in type 2 diabetes which finally leads to hyperglycemia and blood glucose fluctuations [1–3]. Evidence from autopsy also demonstrated that *β*-cell mass in diabetic patients is significantly reduced and its reduction is associated with increased apoptosis [4, 5]. Glucose control may become deteriorated despite intensive treatment in diabetic patients because their pancreatic beta cell mass decreased abnormally. The patients with long duration of diabetes will be prone to show a higher glucose variation [6, 7].

Intermittent high glucose (IHG) as a general phenomenon in the duration of diabetes has been suggested to be more dangerous for the development of diabetes and diabetes-related complications [8, 9]. Several lines of studies showed that blood glucose fluctuations can increase activities of protein kinase C, activate oxidative stress, promote the apoptosis of cell including the *β*-cells, and ultimately result in progressive beta cell failure and beta cell mass reduction [10, 11]. A higher apoptosis of beta cell induced by intermittent high glucose was associated with the increased oxidative stress level [12]. Nevertheless, the mechanism underlying the action of IHG on *β* cells has not yet been fully studied. The previous researches mostly focused on the relationship between apoptosis and IHG; in fact, in addition to cell apoptosis, the lower proliferation activity in *β* cells may be another important reason involved in the *β*-cell mass reduction [13].

In the present study we used an in vitro model to investigate not only the role of IHG in inducing *β*-cells apoptosis but also the effect on proliferation activity in *β*-cells and the possible mechanisms.

## 2. Materials and Methods

### 2.1. Reagents and Antibodies

All reagents for cell culture were purchased from Sigma (St. Louis, MO, USA). Cell Cycle Analysis Kit and Cell counting kit-8 (CCK-8) and Reactive Oxygen Species Assay Kit and Annexin V-FITC Apoptosis Detection Kit were from Beyotime Institute of Biotechnology. Xanthine Oxidase Assay Kit was from BioVision Incorporated (Milpitas, CA, USA). 4, 6-Diamidino-2-phenylindole (DAPI) was purchased from Vector Laboratories (Burlingame, CA). Antibodies against p21 and Skp2 were purchased from Santa Cruz Biotechnology Inc. (Delaware, CA, USA). Alexa Fluor 594-conjugated goat anti-rabbit IgG and cyclinD1 antibodies were purchased from Invitrogen (Carlsbad, CA, USA). Polyclonal antibodies of p27 were purchased from Millipore (Bedford, MA, USA).

### 2.2. Cell Culture

The INS-1 cells were purchased from cell repository of laboratory animal centre in Wuhan University. And then they were grown at 37°C in a humidified 5% CO_2_ atmosphere in RPMI-1640 medium (pH 7.4) supplemented with 10% fetal bovine serum. When the cells were in a synchronization state, cells were cultured, respectively, in RPMI-1640 complete medium with the normal glucose concentration (5.5 mmol/L), constant high glucose concentration (25 mmol/L), and the intermittent high glucose concentration (the rotation per 24 h in 11.1 mmol/L or 25 mmol/L) for 7days.

### 2.3. Determination of Apoptotic Cells

Annexin V-FITC/PI double staining was used to evaluate the cell apoptosis strictly following the procedures of Annexin V-FITC Apoptosis Detection Kit. Briefly, cells were trypsinized and resuspended at a concentration of 1 × 10^6^/mL in dilute binding buffer and labeled with 10 *μ*L of Annexin V-FITC at room temperature in the dark for 30 min; then add 5 *μ*L of PI for 5 min; 400 *μ*L of 1 × binding buffer was added into each tube later.

Flow cytometric analysis was finally performed with excitation at 488 nm as soon as possible to monitor the green fluorescence of Annexin V and the red fluorescence of DNA-bound PI.

### 2.4. Reactive Oxygen Species Production

Intracellular ROS generation was measured by flow cytometry using peroxide-sensitive fluorescent probe 2′,7′-dichlorofluorescein diacetate (DCFH-DA, Molecular Probes) following the method described previously [14]. DCFH-DA will be converted by intracellular esterases to DCFH, a nonfluorescent compound; then it is oxidized into the highly fluorescent dichlorofluorescein (DCF) in the presence of oxidant. In brief, the treated cells were harvested and incubated with 10 *μ*M of DCFH-DA for 30 min at 37°C in the dark, and after being washed twice with PBS, the fluorescent intensity of cells from different groups was analyzed by flow cytometry.

### 2.5. XOD Activity Assay

The XOD activity was measured spectrophotometrically using the Xanthine Oxidase Activity Colorimetric/Fluorometric Assay Kit [15]. One unit xanthine oxidase is defined as the amount of enzyme which catalyzes the oxidation of xanthine, yielding 1.0 *μ*mol of uric acid and H_2_O_2_ per minute at 25°C. Briefly, cells were trypsinized and resuspended at a concentration of 1 × 10^6^/mL with dH_2_O (400 *μ*L), then extracted with 4 volumes of the Assay Buffer, and centrifuged (16,000 ×g, 10 min) to get clear XO extract. For the positive control, add 5 *μ*L positive control solution to wells and adjust volume to 50 *μ*L/well with dH_2_O. For each well, prepare a total 50 *μ*L reaction mix containing 44 *μ*L Assay Buffer, 2 *μ*L substrate mix, 2 *μ*L enzyme mix, and 2 *μ*L OxiRed Probe. Add 50 *μ*L of the reaction mix to each well containing the H_2_O_2_ standard, positive control, and test samples and mix well. Measure the plate immediately (OD = 570 nm for colorimetric assay).

### 2.6. Cell Counting Kit-8 Assay for Proliferation Activity

The treated INS-1 cells were cultured in Corning 96-well flat bottomed microtiter plates for 7 days. 10 *μ*L of CCK-8 was then added and incubated in a high humidity environment at 37°C and 5% CO_2_ for an hour and optical difference (OD) was read at 460 nm with a microplate reader [16]. The OD value represents the proliferation activity.

### 2.7. Cell Cycle Analysis

Cell cycle analysis was carried out by flow cytometry according to a standard protocol. INS-1 cells were gained by centrifugation, washed with cold PBS, and fixed with cold 70% ethanol for 12 hours. Then the fixed cells were stained with PI solution consisting of 50 *μ*g/mL PI, 20 *μ*g/mL RNase A, and 0.1% Triton X-100. After 0.5 h incubation in the dark, the stained cells were detected in a FACScan flow cytometer. The distribution of cells in the different cell cycle phases was analyzed using Multicycle software (Phoenix Flow Systems, San Diego, CA). The ratio of cells in each phase of the cell cycle was determined in triplicate [17].

### 2.8. Western Blotting Analysis

The treated cells were lysed in protein lysis buffer (1% SDS in 25 mmol/L Tris-HCl, pH = 7.4, 1 mmol/L EDTA, 100 mmol/L NaCl, 1 mmol/L PMSF, 10 *μ*g/mL leupeptin, and 10 *μ*g/mL pepstatin). Cell lysates were frozen and thawed 3 times and were further centrifuged at 12,000 g for 10 min at 4°C to pelletize the insoluble material. The protein concentration was measured using the BCA protein assay. The total protein (20 *μ*g) from each sample was separated on 12% SDS-polyacrylamide gel and transferred to polyvinylidene fluoride (PVDF) membranes. Membranes were blocked in 5% nonfat milk and incubated with either anti-cyclinD1 (1 : 200), anti-Skp2 (1 : 100), anti-p21 (1 : 200), anti-p27 (1 : 1000) antibody, or anti-*β*-actin (1 : 2000) antibody overnight. After extensive washing, the immunocomplexes were detected using Western Blotting Luminol Reagent.

### 2.9. Statistical Analysis

Every experiment was repeated five times. All data were expressed as the mean ± S.D. of triplicate experiments. Differences between groups were tested by one-way ANOVA followed by a Student-Newman-Keuls test. And *P* values less than 0.05 were considered as statistically significant.

## 3. Results

### 3.1. Apoptosis in INS-1 Cells Induced by Intermittent High Glucose Measured by Annexin-V/PI Staining

**Figure 1:**
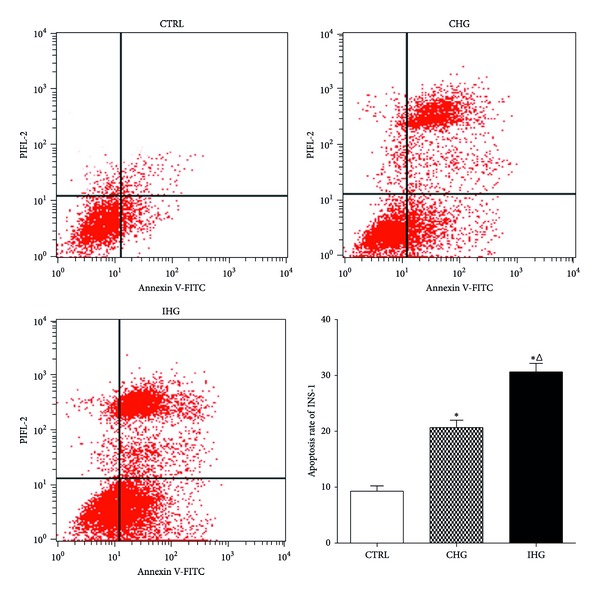
Determination of apoptotic cells with flow cytometry. After the treatment for 7 days, cells were collected for the detection of cell apoptosis as described previously. The percentage of apoptotic cells was shown after analysis by flow cytometry. Data were expressed as mean ± SD of three determinations. Control, normal glucose; CHG, constant high glucose; IHG, intermittent high glucose. *P < 0.05, and ^Δ^ *P* < 0.05 respectively, indicate a significant difference from controls and CHG.

The apoptosis of INS-1 cells was determined by Annexin-V/PI staining via flow cytometry. The percentage of apoptotic cells (Figure 1) in CHG and IHG group was, respectively, 20.92 ± 3.88% and 30.67 ± 4.48%, significantly higher than the control group (normal glucose) 8.91 ± 2.92%. The apoptosis rate induced by IHG showed a statistical difference (*P* < 0.05) compared with the CHG and control group. This result means that intermittent high glucose significantly increased the cell apoptosis compared with both normal and constant high glucose concentrations.

### 3.2. Intermittent High Glucose Increased Intracellular Reactive Oxygen Species Level by Enhancing the Intracellular Xanthine Oxidase Activity

**Figure 2:**
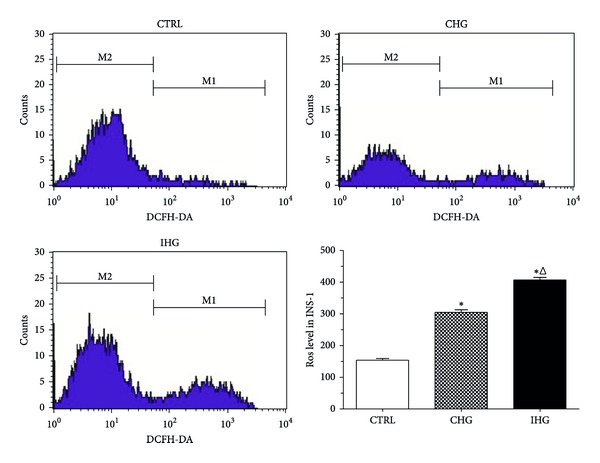
Intracellular reactive oxygen species level of INS-1 cells with DCFH-DA staining. Images of DCFH-DA staining and data of fluorescence intensity analysis for INS-1 cells exposed to different treatment by flow cytometry were demonstrated. Control, normal glucose; CHG, constant high glucose; IHG, intermittent high glucose. *P < 0.05 and ^Δ^ *P* < 0.05, respectively, indicate a significant difference from controls and CHG.

**Figure 3:**
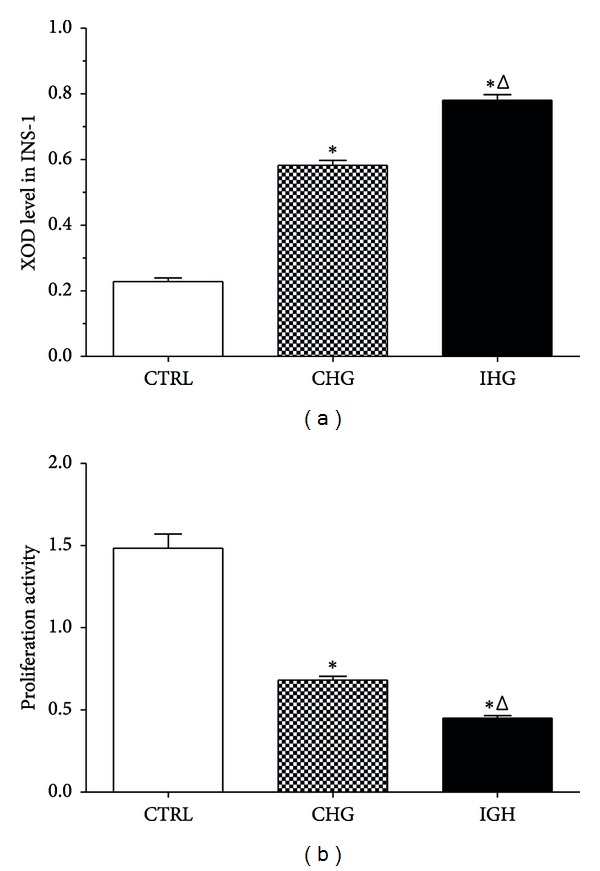
The intracellular XOD activity and cell viability in INS-1 cells. XOD activity was measured spectrophotometrically using Xanthine Oxidase Activity Colorimetric/Fluorometric Assay Kit (a). The cell viability in INS-1 cells was measured by Cell Counting Kit-8 assay (b). Control, normal glucose; CHG, constant high glucose; IHG, intermittent high glucose. Each bar represents the mean ± S.D. *P < 0.05 and ^Δ^ *P* < 0.05, respectively, indicate a significant difference from controls and CHG.

To evaluate the influence of IHG on intracellular levels of oxidative stress, we measured the intracellular reactive oxygen species level by flow cytometry using DCFH-DA fluorescence staining as described previously. As shown in Figure 2, the intracellular reactive oxygen species level in INS-1 cells exposed to CHG increased by 2-fold compared with control (normal glucose), but, the reactive oxygen species level in cells exposed to IHG significantly increased nearly by 2.63-fold compared with control. The intracellular XOD activity was measured spectrophotometrically at the same time. We found that (Figure 3(a)) the cells exposed to CHG showed a significantly increased XOD activity (0.58 ± 0.03) compared with normal glucose group (0.23 ± 0.03), while the cells exposed to IHG had the highest XOD activity (0.79 ± 0.03) and showed a statistical difference (*P* < 0.05).

### 3.3. Effect of Intermittent High Glucose on Proliferation Activity in INS-1 Cells

We evaluated the effect of intermittent high glucose on cell proliferation activity in INS-1 cells with the CCK-8 assay. As shown in Figure 3(b), INS-1 cells treated with CHG showed a significantly reduced cell viability (0.67 ± 0.04) compared with normal glucose group (1.51 ± 0.14). The cells exposed to IHG showed the lowest proliferation activity and the difference was statistical (*P* < 0.05).

### 3.4. Effect of Intermittent High Glucose on Cell Cycle Distribution

**Figure 4:**
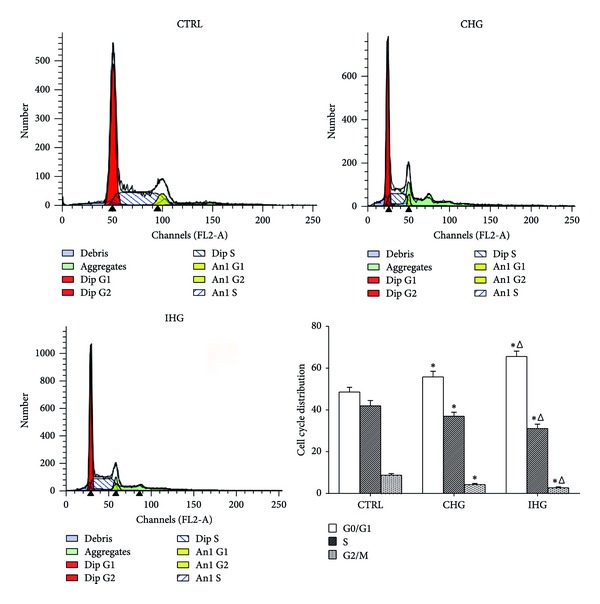
Determination of cell cycle with flow cytometry. After the treatment for 7 days, cells were collected for the detection of cell cycle according to the standard protocol. The distribution of cells in the different cell cycle phases was analyzed using Multicycle software. Data were expressed as mean ± SD of three determinations. Control, normal glucose; CHG, constant high glucose; IHG, intermittent high glucose. *P < 0.05, and ^Δ^ *P* < 0.05 respectively, indicate a significant difference from controls and CHG.

To determine whether IHG regulates the cell cycle of INS-1 cells, the distribution of treated INS-1 cells in various compartments of the cell cycle was examined by flow cytometry. The ratio of cells in each phase of the cell cycle in different groups is summarized in Figure 4. Cells treated with IHG showed a marked accumulation in the G0/G1 phase and decrease in the G2/M phase compared with the normal glucose and CHG group (*P* < 0.05).

### 3.5. Effect of Intermittent High Glucose on Expression of CyclinD1, Skp2, p21, and p27 Protein Levels in INS-1 Cells

**Figure 5:**
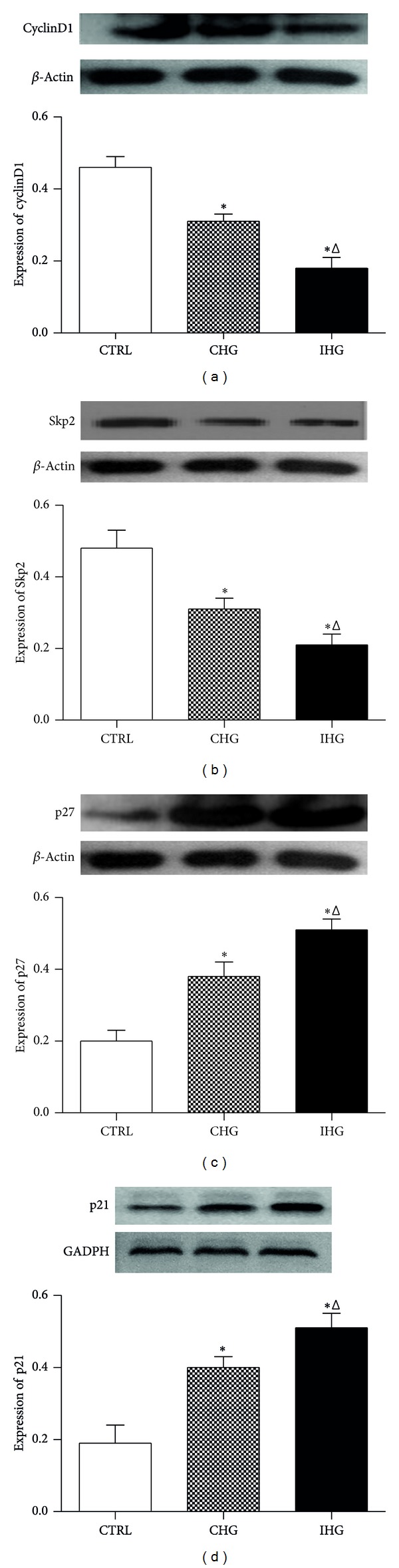
Expressions of cyclinD1, Skp2, p21, and p27 protein level in INS-1 cells were measured by Western blot analyses. Control, normal glucose; CHG, constant high glucose; IHG, intermittent high glucose. Data were expressed as mean ± SD. *P < 0.05 and ^Δ^ *P* < 0.05, respectively, indicate a significant difference from controls and CHG.

To investigate the cellular mechanism by which IHG inhibited the proliferation activity of INS-1 cells, we analyzed the expressions of cyclinD1, Skp2, p21, and p27 protein levels using Western blot analysis. The expression of cyclinD1 analyzed by Western blotting was shown in Figure 5(a); IHG could markedly downregulate the expression of cyclinD1 compared with CHG and normal glucose group (*P* < 0.05). As shown in Figure 5(b), the expression of Skp2 was significantly decreased by IHG (*P* < 0.01). Meanwhile we found that in Figures 5(c) and 5(d) the expressions of p27 and p21 protein level in INS-1 cells were increased by IHG obviously compared with the normal glucose group (*P* < 0.05). These results indicate that IHG may inhibit the proliferation activity of INS-1 cells through regulating the cyclins, such as cyclinD1, Skp2, p21, and p27.

## 4. Discussion

Recently, the apoptosis of pancreatic beta cells has become a major focus. There is a growing body of evidence suggesting that pancreatic beta cell mass in diabetic patients is significantly reduced which mainly resulted from the increased apoptosis. Both animal and human studies have demonstrated that increase of *β*-cell apoptosis is an important reason for insulin deficiency in T2DM, which finally leads to hyperglycemia and blood glucose fluctuations [4, 5, 18]. It is now clear that hyperglycemia is not only the result of *β*-cell deficiency, but also the important reason for further *β*-cell dysfunction and number reduction. Both human pancreatic islets incubated with high glucose concentrations and animal models which received hyperglycemia treatment repeatedly show an obvious increase of apoptosis [19, 20]. Although pancreatic beta cell glucose toxicity is usually characterized as an impairment of beta cell functions induced by chronic constant high glucose, more and more studies have suggested intermittent high glucose (IHG) to be more dangerous for further *β*-cell dysfunction and the development of diabetes. IHG could more easily induce human umbilical vein endothelial cells injury and promote development of diabetic chronic vascular complications compared with sustained high blood glucose (SHG) [21, 22]. IHG can induce a more significant impairment of insulin release response in rat islets and INS-1 cell than SHG; INS-1 cells exposed to IHG showed higher apoptosis and lower GSIS than SHG [23]. In the present study, we similarly found that a higher apoptosis rate of cultured INS-1 cells was induced by IHG compared with CHG and normal glucose. This result is in agreement with previous researches.

There are many potential mechanisms whereby blood glucose fluctuation might cause pancreatic beta cell damage. Blood glucose fluctuations can enhance activity of protein kinase C and NAD(P)H-oxidase and increase oxidative stress, which in turn induce increased apoptosis [10, 11, 24, 25]. In agreement with previous researches, the present study showed that the intracellular oxidative stress was more significantly hyperactivated under the condition of IHG than the condition of CHG. Meanwhile, the intracellular XOD activity in INS-1 cells cultured in IHG was enhanced significantly compared with CHG. We know that Xanthine oxidase (XOD), an enzyme widely distributed in mammal tissues, can catalyze metabolism of hypoxanthine to xanthine and then to uric acid in the presence of molecular oxygen accompanied with O^2−^ generation. XOD level could be upregulated in diabetes and a main source of oxidants [26–28]. These findings suggest that fluctuation in glycemic control could be more deleterious to the pancreatic beta cells than a constant high glucose. The intracellular oxidative stress produced by hyperactivated XOD activity might play a major role in mediating the effect of IHG.

Currently, proliferation from preexisting beta cells is considered to be a major source of newly derived pancreatic beta cells. The extent of beta cell proliferation changes over time according to the metabolic requirements; diabetes occurs when this ability of pancreatic beta cells is attenuated [13, 29, 30]. Hyperglycemia especially the intermittent high glucose that resulted from *β*-cell deficiency can conversely influence the *β*-cell proliferation; it has been proved that IHG could increase both cell apoptosis and inhibition of proliferation in human umbilical vein endothelial cells through NF-*κ*B pathway [22]. The study herein observed the similar effects of IHG on the proliferation activity of INS-1 cells. As shown in Figure 3(b), the cells exposed to IHG showed lower cell viability compared with CHG. We also found that treatment of IHG increased accumulation of INS-1 cells in the G0/G1 phase and notably decreased the ratio of the cells in G2/M phase. Our results demonstrated that the lower proliferation activity and cell cycle arrest induced by IHG could partially be responsible for pancreatic beta cell mass reduction.

It is well documented that cell cycle is controlled by the sequential formation, activation, and inactivation of a series of cell cycle regulators, that is, cyclins, cyclin-dependent kinases (CDK), and cyclin-dependent kinase inhibitors (CKIs). CyclinD1 as positive regulators of cell cycle rises in G1 phrase and remains high until mitosis, Cdks4, 5, and 6 complexes mainly combine with the cyclin D family and function during the G0/G1-phases of the cycle [31]. Cyclin D1 overexpression in beta cells results in a higher rate of beta cell proliferation in vivo, consistent with a similar study performed on rat and human islets in vitro [32, 33]. A number of negative regulators also exist, the Cdk inhibitors (CDKIs), such as the CIP (Cdk-interacting protein)/KIP (kinase inhibitor protein) families including p21, p27 are involved in cyclin binding and kinase inhibitory function. Studies showed that islets from p21cip1-/- mouse treated with mitogens in vitro showed higher DNA synthesis compared with p21-expressing islets. p27 kip1 regulates beta cell proliferation by arresting the cell cycle in the adult [34–36]. The accurately ordered cell cycle also includes the timely degradation of many regulatory proteins via ubiquitin-mediated proteolysis; the SCF complexes represent a big family of ubiquitin ligases that determine the abundance of cell cycle regulatory proteins; Skp2 promotes the ubiquitinylation of the CDKIs, p21, and p27, thereby enhancing CDK2 activation at the G1/S border [37, 38]. Consistent with previous researches, in our study, the cells with low cell viability induced by IHG showed a decreased expression of cyclinD1 and Skp2 and an increased expression of p27 and p21. The cell cycle of INS-1 cells was blocked mainly in G0/G1 phrase.

In summary, our study simultaneously investigated the apoptosis-inducing and antiproliferative role of IHG on INS-1 cells and preliminarily revealed the possible mechanism. Chronic exposure to IHG may not only result in more effective induction of apoptosis via increasing intracellular oxidative stress produced by hyperactivated XOD activity, but also inhibit the cell viability and block the cell cycle progression through changing the expressions of cell cycle regulators. We know that the cell signal transduction pathways are more multifarious and complicated; further studies may be needed for understanding the exact signaling and mechanism, especially studies based on primary *β*-cells and the in vivo studies.

## References

[1] Donath MY, Ehses JA, Maedler K (2005). Mechanisms of *β*-cell death in type 2 diabetes. *Diabetes*.

[2] Prentki M, Nolan CJ (2006). Islet *β* cell failure in type 2 diabetes. *Journal of Clinical Investigation*.

[3] Rhodes CJ (2005). Type 2 diabetes—a matter of *β*-cell life and death?. *Science*.

[4] Butler AE, Janson J, Bonner-Weir S, Ritzel R, Rizza RA, Butler PC (2003). *β*-cell deficit and increased *β*-cell apoptosis in humans with type 2 diabetes. *Diabetes*.

[5] Yoon KH, Ko SH, Cho JH (2003). Selective *β*-cell loss and *α*-cell expansion in patients with type 2 diabetes mellitus in Korea. *Journal of Clinical Endocrinology and Metabolism*.

[6] UK Prospective Diabetes Study (UKPDS) Group (1998). Intensive blood-glucose control with sulphonylureas or insulin compared with conventional treatment and risk of complications in patients with type 2 diabetes (UKPDS 33). *The Lancet*.

[7] Murata GH, Duckworth WC, Shah JH, Wendel CS, Hoffman RM (2004). Sources of glucose variability in insulin-treated type 2 diabetes: the Diabetes Outcomes in Veterans Study (DOVES). *Clinical Endocrinology*.

[8] Piconi L, Corgnali M, Da Ros R, Assaloni R, Piliego T, Ceriello A (2008). The protective effect of rosuvastatin in human umbilical endothelial cells exposed to constant or intermittent high glucose. *Journal of Diabetes and its Complications*.

[9] Ceriello A, Ihnat MA (2010). ’Glycaemic variability’: a new therapeutic challenge in diabetes and the critical care setting. *Diabetic Medicine*.

[10] Muggeo M, Zoppini G, Bonora E (2000). Fasting plasma glucose variability predicts 10-year survival of type 2 diabetic patients: the Verona Diabetes Study. *Diabetes Care*.

[11] Kohnert KD, Freyse EJ, Salzsieder E (2012). Glycaemic variability and pancreatic *β*-cell dysfunction. *Current Diabetes Reviews*.

[12] Hou ZQ, Li HL, Gao L, Pan L, Zhao JJ, Li GW (2008). Involvement of chronic stresses in rat islet and INS-1 cell glucotoxicityn induced by intermittent high glucose. *Molecular and Cellular Endocrinology*.

[13] Yesil P, Lammert E (2008). Islet dynamics: a glimpse at beta cell proliferation. *Histology and Histopathology*.

[14] Sheu ML, Ho FM, Yang RS (2005). High glucose induces human endothelial cell apoptosis through a phosphoinositide 3-kinase-regulated cyclooxygenase-2 pathway. *Arteriosclerosis, Thrombosis, and Vascular Biology*.

[15] Tamta H, Kalra S, Mukhopadhyay AK (2006). Biochemical characterization of some pyrazolopyrimidine-based inhibitors of xanthine oxidase. *Biochemistry*.

[16] Mwitari PG, Ayeka PA, Ondicho J, Matu EN, Bii CC (2013). Antimicrobial activity and probable mechanisms of action of medicinal plants of Kenya: withania somnifera, Warbugia ugandensis, Prunus africana and Plectrunthus barbatus. *PLOS ONE*.

[17] Zhang T, Tan Y, Zhao R, Liu Z (2013). DNA damage induced by oridonin involves cell cycle arrest at G2/M phase in human MCF-7 cells. *Contemporary Oncology*.

[18] Shi XL, Ren YZ, Wu J (2011). Intermittent high glucose enhances apoptosis in INS-1 cells. *Experimental Diabetes Research*.

[19] Mohanty S, Spinas GA, Maedler K (2005). Overexpression of IRS2 in isolated pancreatic islets causes proliferation and protects human *β*-cells from hyperglycemia-induced apoptosis. *Experimental Cell Research*.

[20] Maedler K, Schulthess FT, Bielman C (2008). Glucose and leptin induce apoptosis in human *β*-cells and impair glucose-stimulated insulin secretion through activation of c-Jun N-terminal kinases. *FASEB Journal*.

[21] Li H, Télémaque S, Miller RE, Marsh JD (2005). High glucose inhibits apoptosis induced by serum deprivation in vascular smooth muscle cells via upregulation of Bcl-2 and Bcl-xl. *Diabetes*.

[22] Chen G, Chen Y, Chen H (2011). The effect of NF-*κ*B pathway on proliferation and apoptosis of human umbilical vein endothelial cells induced by intermittent high glucose. *Molecular and Cellular Biochemistry*.

[23] Kim M, Chung H, Yoon C (2012). Increase of INS-1 cell apoptosis under glucose fluctuation and the involvement of FOXO-SIRT pathway. *Diabetes Research and Clinical Practice*.

[24] Sun LQ, Chen YY, Wang Li XJ X (2012). The protective effect of alpha lipoic acid on Schwann cells exposed to constant or intermittent high glucose. *Biochemical Pharmacology*.

[25] Del Guerra S, Grupillo M, Masini M (2007). Gliclazide protects human islet beta-cells from apoptosis induced by intermittent high glucose. *Diabetes/Metabolism Research and Reviews*.

[26] Gibbings S, Elkins ND, Fitzgerald H (2011). Xanthine oxidoreductase promotes the inflammatory state of mononuclear phagocytes through effects on chemokine expression, peroxisome proliferator-activated receptor-*γ* sumoylation, and HIF-1*α*. *Journal of Biological Chemistry*.

[27] Kushiyama A, Okubo H, Sakoda H (2012). Xanthine oxidoreductase is involved in macrophage foam cell formation and atherosclerosis development. *Arteriosclerosis, Thrombosis, and Vascular Biology*.

[28] Miric D, Kisic B, Stolic R, Miric B, Mitic R, Janicijevic-Hudomal S (2013). The role of xanthine oxidase in hemodialysis-induced oxidative injury: relationship with nutritional status. *Oxidative Medicine and Cellular Longevity*.

[29] Dor Y, Brown J, Martinez OI, Melton DA (2004). Adult pancreatic *β*-cells are formed by self-duplication rather than stem-cell differentiation. *Nature*.

[30] Garcia-Ocaña A, Alonso LC (2010). Glucose mediated regulation of beta cell proliferation. *The Open Endocrinology Journal*.

[31] Bicknell KA, Surry EL, Brooks G (2003). Targeting the cell cycle machinery for the treatment of cardiovascular disease. *Journal of Pharmacy and Pharmacology*.

[32] Cozar-Castellano I, Takane KK, Bottino R, Balamurugan AN, Stewart AF (2004). Induction of *β*-cell proliferation and retinoblastoma protein phosphorylation in rat and human islets using adenovirus-mediated transfer of cyclin-dependent kinase-4 and cyclin D1. *Diabetes*.

[33] Zhang X, Gaspard JP, Mizukami Y, Li J, Graeme-Cook F, Chung DC (2005). Overexpression of cyclin D1 in pancreatic *β*-cells in vivo results in islet hyperplasia without hypoglycemia. *Diabetes*.

[34] Pines J (1997). Cyclin-dependent kinase inhibitors: the age of crystals. *Biochimica et Biophysica Acta—Reviews on Cancer*.

[35] Cozar-Castellano I, Weinstock M, Haught M, Velázquez-Garcia S, Sipula D, Stewart AF (2006). Evaluation of *β*-cell replication in mice transgenic for hepatocyte growth factor and placental lactogen: comprehensive characterization of the G1/S regulatory proteins reveals unique involvement of p21 cip. *Diabetes*.

[36] Karnik SK, Hughes CM, Gu X (2005). Menin regulates pancreatic islet growth by promoting histone methylation and expression of genes encoding p27Kip1 and p18INK4c. *Proceedings of the National Academy of Sciences of the United States of America*.

[37] Carrano AC, Eytan E, Hershko A, Pagano M (1999). SKP2 is required for ubiquitin-mediated degradation of the CDK inhibitor p27. *Nature Cell Biology*.

[38] Bornstein G, Bloom J, Sitry-Shevah D, Nakayama K, Pagano M, Hershko A (2003). Role of the SCFSkp2 ubiquitin ligase in the degradation of p21Cip1 in S phase. *Journal of Biological Chemistry*.

